# Inflammasome Fuels Dengue Severity

**DOI:** 10.3389/fcimb.2020.00489

**Published:** 2020-09-10

**Authors:** Gaurav Shrivastava, Paola Carolina Valenzuela Leon, Eric Calvo

**Affiliations:** Laboratory of Malaria and Vector Research, National Institute of Allergy and Infectious Diseases, National Institutes of Health, Rockville, MD, United States

**Keywords:** dengue (DENV), NLRP3 inflammasome, innate immune response, cytokine storm, IL-1β, mosquito borne disease, vascular leakage, pyroptosis

## Abstract

Dengue is an acute febrile disease triggered by dengue virus. Dengue is the widespread and rapidly transmitted mosquito-borne viral disease of humans. Diverse symptoms and diseases due to Dengue virus (DENV) infection ranges from dengue fever, dengue hemorrhagic fever (life-threatening) and dengue shock syndrome characterized by shock, endothelial dysfunction and vascular leakage. Several studies have linked the severity of dengue with the induction of inflammasome. DENV activates the NLRP3-specific inflammasome in DENV infected human patients, mice; specifically, mouse bone marrow derived macrophages (BMDMs), dendritic cells, endothelial cells, human peripheral blood mononuclear cells (PBMCs), keratinocytes, monocyte-differentiated macrophages (THP-1), and platelets. Dengue virus mediated inflammasome initiates the maturation of IL-1β and IL-18, which are critical for dengue pathology and inflammatory response. Several studies have reported the molecular mechanism through which (host and viral factors) dengue induces inflammasome, unravels the possible mechanisms of DENV pathogenesis and sets up the stage for the advancement of DENV therapeutics. In this perspective article, we discuss the potential implications and our understanding of inflammasome mechanisms of dengue virus and highlight research areas that have potential to inhibit the pathogenesis of viral diseases, specifically for dengue.

## Introduction

Dengue fever is an acute febrile disease triggered by the dengue virus (DENV). DENV is positive sense, single stranded, RNA virus, belongs to the *Flaviviridae* family, genus *flavivirus*. *Aedes* mosquitoes, *Aedes aegypti* and occasionally *Aedes albopictus* transmit DENV to humans. The disease is caused by mainly four DENV serotypes, DENV 1-4. DENV imposes risk for more than 2.5 billion people with likely 50 million infections per year of infection, majorly in tropical and subtropical countries (Guha-Sapir and Schimmer, 2005; Brady et al., 2012; Bhatt et al., 2013). Constant circulation of different serotypes of the virus further leads to cross-reactivity and imposes added risk on successive dengue infections (secondary infection). DENV infection leads to several clinical symptoms including dengue fever (DF) that is asymptomatic mild flu, to coagulopathy, enhanced vascular fragility, and thrombocytopenia, well-known as Dengue Hemorrhagic fever (DHF), that lead to hypovolemic shock called Dengue Shock Syndrome (DSS), a more severe condition (Murphy and Whitehead, 2011; Afroz et al., 2016). Several factors contribute to DENV pathogenesis such as substantial cytokine secretion (“cytokine storm”), host genetics, heterotypic secondary infection, and antibody-dependent enhancement (ADE) (Pang et al., 2007; Katzelnick et al., 2017). Although, dengue pathogenesis remains elusive, the cytokine storm has been considered to be one of the primary and crucial causative factors (Hatch et al., 2011; Rothman, 2011). IL-1β, a very strong cytokine that is intensely regulated and stimulated by DENV infected macrophages and monocytes, considered as an important component in cytokine storm (Chang and Shaio, 1994; Dinarello, 2009; Wu et al., 2013b). Several evidences have demonstrated the elevated level of IL-1β in serum cytokine and gene expression profiles in severe dengue patients suggesting the contribution of IL-1β in the severity of dengue pathology (Bozza et al., 2008; Jaiyen et al., 2009). IL-1β increases the vascular permeability, especially in concurrence with TNF-α and IFN-β in many severe dengue profiles (Netea et al., 2000; Dinarello, 2004). As a part of its role as an endogen pyrogen (fever-causing), IL-1β stimulates lymphocytes and foster leukocytes infiltration to the inflammation site, thereby regulating local and systemic inflammation (Dinarello, 2011). Two-step activation pathways are required in the production of IL-1β: “priming signal” fuels the induction of transcription and synthesis of pro-IL-1β, and subsequent “activation signal” assembles and activates inflammasome and caspase-1 (Schroder and Tschopp, 2010). “Inflammasomes” are multimeric protein complexes of innate immune system that form in the cytosol after detecting PAMPs (pathogen associated molecular patterns) or DAMPs (damage associated molecular patterns). Inflammasome protein complex comprises of a sensor proteins; NLR [nucleotide-binding domain (NBD) and leucine-rich-repeat-(LRR)-containing] family, AIM2 like receptor, ALR, an adaptor protein ASC (apoptosis-associated speck-like protein containing a CARD), and an inactive zymogen, procaspase-1 (Martinon et al., 2002; Rathinam et al., 2012). Activation of inflammasomes in response to PAMP and DAMP signals leads to autocleavage process, which in turn process pro-caspase-1 to active caspase-1. Activated caspase-1 then converts pro-IL-1β and pro-IL-18 into their active forms (IL-1β and IL-18, respectively) (Martinon et al., 2002). Inflammasome plays a vital function in the host innate immune system by regulating the secretion of proinflammatory cytokines (IL-1β and IL-18) and subsequent induction of “pyroptosis,” a form of programmed cell death, activated by inflammatory caspases (Fink and Cookson, 2006; Lamkanfi and Dixit, 2014). Although a variety of pathogen senses the PAMP and DAMPs and activate diverse inflammasomes, study shows a range of virus infection that activates inflammasomes such as the NLRP3, AIM2, and RIG-I and mediate the robust host immune response (Shrivastava et al., 2016). Inflammasomes protect the host from the attack of microbial pathogens and endogenous danger signals, thereby playing a crucial role in host defense. Irrespective of the location in cytosol, inflammasome creates an efficient immune response against extracellular and intra-cellular foreign invaders. Although the optimum inflammasome activation is extremely favorable to the security of the host, dysregulation of inflammasome activation can leads to the worsening of symptoms in infectious diseases and results in the autoimmune and inflammatory disorders development (Lamkanfi and Dixit, 2014). It has been shown that increased serum levels of IL-1β and IL-18 correlates with the gravity of dengue infection (Mustafa et al., 2001; Bozza et al., 2008). This finding suggests that inflammasome play a critical role in the pathogenesis of dengue infection. In this perspective, we will discuss the inflammasomes role during DENV infection and the mechanism through which inflammasome imparts a central role in dengue severity.

## Virus-Induced Host Inflammasome

Numerous studies have emphasized the significance of inflammasome activation in the regulation of virus infection. A range of upstream sensing receptors comprising the AIM2-like receptor, the NOD-like receptor, and the RIG-I-like receptor determine the inflammasome activation during viral infection. These receptors regulate inflammasome activation while present in different cellular compartments i.e., cytoplasm and the nucleus (Schroder and Tschopp, 2010). Among all the known inflammasomes, the NLRP3 inflammasome is the most significantly studied in viral infections and suggested to play a decisive role in both inflammation and antiviral responses (Chen and Ichinohe, 2015). Viral components as well as several stimuli by virus infection are sensed by host innate immune system and activate NLRP3 inflammasome. There are several stimuli which induce cytosolic DAMP signals, such as protein aggregates, mitochondria injury, and aberrant ion mobilization. Two-step process is prerequisite to trigger the NLRP3 inflammasome. The priming step: TLR, NLR, or RIG-I-like receptor families, IFNAR or by a cytokine receptor activate the priming step that leads to transcriptional upregulation of NLRP3, pro-caspase-1, pro-IL-1β, and pro-IL-18. The activation step: signal for inflammasome activation is triggered in response to infection, tissue damage, metabolic imbalances or diverse stress signals coupled with host sterile cell/tissue damage. Stress signal include pore-forming toxins, nucleic acids, Ca^++^ mobilization, K^+^ efflux, ATP lysosomal damage, crystalline substances and invading pathogens (Lamkanfi and Dixit, 2014; Swanson et al., 2019). Several studies have demonstrated that NLRP3 senses the known activators indirectly due to their structural variability (Bauernfeind et al., 2009; Latz, 2010). Structurally, NLRP3 Inflammasome is made of NLRP3, ASC (adaptor protein apoptosis-associated speck-like protein containing CARD) and procaspase-1. Structurally, NLRP3 has three components i.e., PYD–NOD–LRRs in the order of its domains from N-terminus to C-terminus. During NLRP3 Inflammasome activation, NOD domain arranges the NLRP3 as a homo-oligomer and further links with ASC through its PYD domain. ASC binds with procaspase-1 through its CARD domain to form the complete structure. Thus, NLRP3 inflammasome activation leads to the activation of caspase-1 and secretion of mature IL-1β and IL-18 (Latz, 2010). Subsequently, IL-1β potentially recruits neutrophils to the site of inflammation to assist in the removal of infecting viruses (Niu et al., 2019). Furthermore, IL-1β and IL-18 are also involved in the consequent initiation of the adaptive immune response (Dinarello et al., 2013; Joosten et al., 2013). Although the NLRP3 inflammasome provides the host antiviral status, irregular NLRP3 inflammasome activation leads to severity of pathological injury during virus infection. As an example, in an Influenza A virus (IAV) infection model, NLRP3 inflammasome activation in juvenile mice leads to severe lung injury independent of viral titer, while sustained elevated levels of type I IFNs exist (Coates et al., 2018). IAV infection also shows the high mortality in IL-1RI^−^/^−^ mice as compared to wild type mice (Schmitz et al., 2005). Likewise, IL-18^−^/^−^ shows mortality with increased viral load as compared to wild type mice (Liu et al., 2004). In Herpes simplex virus 1 (HSV-1) infection, increased viral load was observed in IL-1β^−^/^−^ mice as compared to wild-type mice with decreased immune response (Sergerie et al., 2007). In other studies, IL-18 administration prior to HSV-1 infection protect the HSV-1-infected mice and increase survival (Fujioka et al., 1999), revealing the immune control mechanism of IL-1β and IL-18 against the HSV-1 infection. On the other hand, IL-1β and IL-18 protect the host against HSV-1-induced encephalitis. In addition, NLRP3 inflammasome mediates the chronic intrahepatic inflammation and liver injury during HCV infection (Negash et al., 2013). Inflammasome leads to immunopathology progress and recruits excessive inflammatory cells that leads to pyroptosis mediated cell death (McAuley et al., 2013; Haque et al., 2016). Numerous studies have established the activation of NLRP3 inflammasome by viruses with *in vivo* relevance for control of virus infection (Chen and Ichinohe, 2015; Zhao and Zhao, 2020). Viral pathogens such as Encephalomyocarditis virus (ECMV), Hepatitis C virus (HCV), Sendai virus, Human immunodeficiency virus-1 (HIV-1), Vesicular stomatitis virus (VSV), Human chikungunya virus (CHIKV), Japanese encephalitis virus, Respiratory syncytial virus (RSV), Human rhinovirus (HRV), Zika virus (ZIKV), Influenza A virus (IAV), West Nile virus (WNV), Dengue virus (DENV), Rift Valley fever virus (RVFV), and Coronavirus (SARS-CoV) have been reported to activate inflammasomes (Lupfer et al., 2015; Shrivastava et al., 2016; Zhao and Zhao, 2020). Thus, the outcomes of NLRP3 inflammasome activation are very much virus specific (*in vivo* and in clinical settings) and deliver substantial defense against some pathogenic viruses however can also play a detrimental role in immunopathology of others.

## Response of Host Inflammasome To DENV Infection

Dengue infected patients shows a plethora of clinical manifestations including sudden-onset fever, hepatosplenomegaly, headache, muscle and joint pain (Setiawan et al., 1998; Wu et al., 2004). Endogen pyrogens (EP) mediate the fever onset which suggests that DENV may trigger high amounts of endogenous pyrogens (e.g., IL-1β and TNF-α) and lead to high fever onset in the patients.

### Macrophages Induced Inflammasome

Studies have demonstrated that macrophages (Mϕ) serve as major target cells for DENV infection, replication and the major source of inflammatory cytokines (Chen and Wang, 2002; Jessie et al., 2004; Balsitis et al., 2009). Mϕ are heterogeneous in nature and during DENV infection, M-Mϕ (resting macrophages) and GM-Mϕ (inflammatory macrophages) display distinctive cytokines expression profiling and innate immunity receptors/sensors. High expression of CLEC5A, mannose receptor, and NLRP3 were found in GM-Mϕ as compare to M-Mϕ, supporting the hypothesis that these two subsets display distinct functions (Wu et al., 2013a). In the continuation, a striking study has suggested that the functions of human macrophage subsets and corresponding signaling events are instrumental in the production of IL-1β and IL-18. This study demonstrated that CLEC5A serves as a myeloid PRR for DENV, and DENV replicates more competently in GM- Mϕ (inflammatory macrophages) than in M-Mϕ (resting macrophages) (Wu et al., 2013b). DENV infection activates the NLRP3 inflammasome in GM-Mϕ that results in caspase-1 activation and subsequent IL-1β and IL-18 release. Interestingly, DENV infection in GM-Mϕ induces the increased expression of NLRP3 without affecting other sensors i.e., NLRC4 and NLRP1, while siRNA targeted to NLRP3 obstructs DENV stimulated IL-1β and IL-18 secretion. Additionally, DENV infection in GM-Mϕ leads to increased level of TNF-α with decreased level of IL-10. Moreover, released cytokines increase body temperature and stimulate the release of soluble factors that result in vascular permeability during dengue infection (Wu et al., 2013b). These results suggest the important function of CLEC5A in dengue severity as another study also shows that CLEC5A is critical for DHF and DSS (Chen et al., 2008). The study also indicated the crucial role that CLEC5A plays in NLRP3 activation, as blockade of CLEC5A blocks the NLRP3 inflammasome activation, and subsequent ablated DENV induced IL-1β production and caspase-1 mediated proptosis in GM-Mϕ (Wu et al., 2013b). Apart from facilitating innate immune response, IL-1β and IL-18 display a crucial function in fostering adaptive immune response during DENV infection. IL-1β secretion further enhances the production and release of IL-23 and IL-6 and the association of IL-18, IL-1β, and IL-23 stimulate Th17/γδ T cells to generate pro-inflammatory cytokines (GM-CSF, IL-17A, IL-17F, IL-22,) that create the stage for host adaptive immune responses during DENV infection (Wu et al., 2013a). Thus, the macrophage is also considered as the primary target of DENV since DENV induced thrombocytopenia releases IL-18 via the activation of NLRP3 Inflammasome. Epidemiologic study also supports this notion as the severity of DF/DHF were correlated with elevated level of GM-CSF in the plasma of DENV patients (Bozza et al., 2008). This study indicates the probable switch mechanism of resting macrophages to inflammatory macrophages by GM-CSF during DENV infection, that affects the gravity of clinical outcomes. This warrants further investigation as the inhibition of CLEC5A may diminish the severity of dengue induced clinical symptoms in patients. Notably, a study demonstrated the importance of immunosuppressive status based on different age group that influences the differential induction of IL-1β by monocytes during DENV infection (Valero et al., 2014). Monocytes/macrophages from neonatal, adult, and elderly was infected with all four DENV types and cytokine profile was analyzed. The study observed the highest cytokine production (TNF-α, IL-6, and IL-1β) by adult monocytes as compare to neonates and elderly subjects depicting the presence of immunosuppressive condition at both sides of life and may be due to different physio pathological mechanisms (Valero et al., 2014).

### Dendritic Cells and Inflammasomes

DENV also targets, human dendritic cells (DCs), a proficient antigen-presenting cell with immune sensors that mounts the immune response in the peripheral blood and in the lymphoid (Ho et al., 2001) and trigger IFN signaling (Hsu et al., 2016). It has been reported that TLR9 in endolysosomes in DC senses “self-DNA,” binds to it and triggers signaling events, including NF-κB and mitogen- activated protein kinase (MAPK) p38 signaling pathways (Sasai et al., 2010). Mitochondria plays a crucial role in TLR9 mediated pathways as DAMPs released from mitochondria during tissue injury, stress conditions, and cytokine production due to trauma, trigger inflammatory responses through activation of the TLR9 signaling pathway (Zhang et al., 2010; Caielli et al., 2016; Lood et al., 2016). mtDNA that serves as a DAMP, engages several pattern-recognition receptors present on diverse cell types and initiates potent inducement of the proinflammatory immune response and IFN production (West and Shadel, 2017). Another study demonstrated that DENV infects DC and induces NLRP3 inflammasome activation, triggering ROS production in mitochondria that leads to the discharge of mitochondrial DNA (mtDNA) into the cytosol. mtDNA further triggers the production of interferons (IFNs) by activating TLR9 signaling pathways (Lai et al., 2018). These incidents results in the productions of several anti-viral cytokines IFNs, such as IFN-λ3, IFN-λ2, IFN-λ1. Inhibition of inflammasome diminished the release of mtDNA in the cytosol during DENV infection subsequent TLR9 activation. These data support the crucial roles of DENV-induced inflammasome activation and further DENV induced mtDNA-TLR9 axis mediated IFN production and their following incidents.

### DENV Induced Inflammasome in Platelets

During DENV infection, increased IL-1β also correlates with thrombocytopenia (Bozza et al., 2008). A study reports that DENV infects the platelets via DC-SIGN receptor that further leads to platelets activation, mitochondrial dysfunction and activation of the apoptosis caspase cascade, that leads to the development of thrombocytopenia in patients with dengue (Bozza et al., 2008). Another study has tested the hypothesis, whether platelets activation by dengue induce the synthesis and processing of IL-1β, that can be capable of disrupting the endothelial cell barrier function. DENV infection activate the NLRP3 inflammasome, and subsequent activation of caspase-1, and caspase-1 dependent secretion of IL-1β in platelets microparticles from patients with dengue as well as when platelets get exposed to DENV *in vitro*. DENV induced inflammasome activation and subsequent platelet released IL-1β-rich microparticles associated with increased vascular permeability and this increase were found to be IL-1β dependent. In addition, RIP1/RIP3-mediated mitochondrial ROS generation are required for NLRP3 inflammasome activation and subsequent release of mature IL-1β cytokine (p17) and the shedding of IL-1β MPs, indicating the role of RIP1/RIP3-mediated mitochondrial ROS generation in dengue vasculopathy (Hottz et al., 2013).

### Neutrophils Activation, NETs Formation, and Inflammasome

A new mechanism has been demonstrated regarding the role of platelets in DHF. The study demonstrated that DENV activates mouse and human platelets via the CLEC-2 receptor, thereby triggering the release of extracellular vesicles (EVs), including microvesicles (MVs) and exosomes (EXOs). Further, EXOs (DV-EXOs) and MVs (DV-MVs) upregulate the CLEC5A and TLR2 on neutrophils and macrophages, resulting in the formation of a neutrophil extracellular trap (NETs) and subsequent release of proinflammatory cytokine. *In vivo* mouse model, DENV induced NETs formation and inflammasome activation was impaired in CLEC5A^−/−^ and TLR2^−/−^ mouse neutrophils. Furthermore, DENV triggered systemic vascular permeability and lethality were reduced dramatically. Further, *in vivo* study demonstrated the role of NETs in complex interface among macrophages, platelets, and neutrophils during DENV infection as DNase I mediated exclusion of NETs provided certain defensive effect in *stat1*^−/−^ mice (35% survival). Furthermore, DNase I has no effect in the survival of DENV-infected *stat1*^−/−^*clec5a*^−/−^ mice, suggesting the NETs formation induced pathogenic effect in DENV infection by increasing systemic vascular permeability (Sung et al., 2019). NETs induce the interruption of vascular endothelial cell layer and vascular leakage in dengue infection, due to the presence of metalloproteinases and histones in the NETs (Saffarzadeh et al., 2012; Carmona-Rivera et al., 2015). NETs formation outcome varies greatly in virus to virus infections and specific mechanism for NETs formation and the factors that decide the functions of NETs in viral infection are still elusive. Specifically, while NETs show their beneficial roles in preventing viral dissemination (murine pox virus) (Jenne et al., 2013) and trapping (HIV) (Saitoh et al., 2012), formation of disproportionate NETs turn in exacerbated allergic airway inflammation during rhinovirus (RV) infection (Toussaint et al., 2017) and airway obstruction during respiratory syncytial virus (RSV) infection (Cortjens et al., 2016). Another *in vivo* study also demonstrated the neutrophil activation and neutrophil extracellular trap (NETs) formation during an acute DENV infection. *In vitro* incubation of NETs results in reduced DENV infectivity. Notably, NETs components were more predominantly present in the serum patients with hemorrhagic fever (DHF), but not acute phase of the infection (Opasawatchai et al., 2019). Other evidence that demonstrates the excessive inflammation during DENV infection indicates that neutrophils activation and NETs formation can contribute significantly in disease pathogenesis (Costa et al., 2013). This suggest that somehow NETs are inhibited during acute phase, however, in the more severe phase, NETs formation is induced, implying the dual roles of NETs in DENV infection. Although, the NETs formation directly by DENV particle is still debatable due to different views from different studies. Where, in a study, NETs formation has shown to be found in healthy neutrophils during DENV infection (Yost et al., 2016), another study suggests the inhibition of NETs formation due to DENV-2 (Moreno-Altamirano et al., 2015). There could be a link of inflammasome and neutrophil activation and NETs formation, as a study found the overexpression of CD66b in neutrophils, a marker of granulocytes activation and increased ROS production during DENV infection. As ROS production is directly linked to trigger inflammasome, the study observed the increased ROS production in granulocytes, in response to *ex vivo* PMA stimulation during acute dengue infection. This phenomenon is suggested due to TNFα and IL-8 cytokines that present during acute DENV infection. ROS is instrumental in ROS-dependent NETs formation, neutrophil antimicrobial activity and inflammasome activation (Opasawatchai et al., 2019). Another independent study demonstrates the role of GSDMD (Gasdermin D), a pore-forming protein and an initiator of pyroptosis with NETs formation. It has been reported that in non-canonical inflammasomes activation, neutrophils trigger the release of NETs and it was found to be GSDMD dependent. It is reported that neutrophils get several unknown stimuli that activate the protease that further cleaves GSDMD to implement NETosis. Furthermore, ROS is required during classical NETosis that activates serine proteases that too cleave GSDMD (Chen et al., 2018). Further, GSDMD acts as a feed forward loop and includes protease activation and nuclear expansion (Sollberger et al., 2018). How these mechanisms exist during DENV infection is not known and more research understanding regarding the neutrophil activation, NETs formation, inflammasome activation and their interconnection functions are warranted. Overall, this data unravels the central role of CLEC5A/TLR2 and CLEC2 in “neutrophil–platelet interactions” that fuels the enhanced inflammatory reactions during DENV infection (Sung et al., 2019).

### Inflammasome Stages the Adaptive Immune Response

Another study emphasizes the inflammasome role in mounting the immune response in dengue virus infection. It has demonstrated that primary human γδ T cells (freshly isolated) react promptly to DENV infected DC (dendritic cells) and produces IFN-γ and upregulates CD107a. This anti-DENV IFN- γ is controlled by DENV infected DC that induces type 1 IFN and IL-18 (Tsai et al., 2015). To note, dengue infected patients plasma demonstrated the elevated levels of type I IFN and IL-18 (Mustafa et al., 2001; Gandini et al., 2013) and these cytokines (type I IFN and IL-18) by producing IFN-γ by γδ T display a physiologically phenomenon in fostering an effective anti-DENV Th1 adaptive-immune response and an important determinant of dengue disease severity. Further, the inhibition of inflammasome activation (due to extracellular ATP) weakened the IFN-γ response of γδ T cells. Notably, monocytes serve as an accessory cells as it induces the IL-18 that further triggers the γδ T cells during infected DC response to DENV (Tsai et al., 2015). A wide range of cells shows the activation and expression of both CC and CXC chemokines due to monocytes/macrophages induces IL-18 that augment neutrophil activity, IFN-γ production and natural killer (NK) cell cytotoxicity by NK cells (Dinarello et al., 2013). Therefore, to gain the full anti DENV activity of γδ T cells, two activation checkpoints are required: Secretion of type I IFN through IRF-dependent pathways (e.g., detection of viral RNA by TLR3 and RIG-like receptors) (Loo et al., 2008; Nasirudeen et al., 2011) and the recognition of released extracellular ATP (due to cell damage) lead to activation of inflammasome (Dinarello and Fantuzzi, 2003).

### Inflammatory Mediators of Dengue Disease Biomarker

Monocytes activation and aberrant inflammasome activation have demonstrated a central role in the advancement of severe form of dengue disease that also serves as a biomarker of dengue patients. Recently, a study investigated 20 plasma biomarkers from dengue virus infected patient including chemokines, cytokines, along with additional inflammatory mediators that forecast the severity of dengue disease according to the WHO 2009 classification. This study identified IL-18, LBP (LPS binding protein) and sCD14 as a best predictive value, particularly at the febrile phase as their presence is significantly higher than any other marker tested (Yong et al., 2017). The phenomenon of microbial translocation (MT) has been described where LPS translocate from gut into the blood stream under inflammatory conditions. Microbial translocation play a vital role in HIV disease (Brenchley et al., 2006), inflammatory bowel syndrome (Rojo et al., 2007), chronic liver disease (Pinzone et al., 2012) and even end stage kidney disease (Wang et al., 2012). More recently, MT has been reported among dengue patients (van de Weg et al., 2012) and levels of LPS were accompanied with progression of dengue disease as LPS has been found to act along with DENV in triggering IL-6, PAF, and TNF-α (van de Weg et al., 2013; Kamaladasa et al., 2016). In the context of LBP, LBP is a soluble acute phase protein that initiates immune responses by binding directly to bacterial LPS and relocate it to monocyte/macrophages expressing sCD14 and TLR4 on cell surface (Opal et al., 1999), as LPS stimulation to monocyte and macrophages triggers sCD14 secretion (Landmann et al., 1995). The study, therefore, advocates the central role of MT in intensifying the inflammatory response in dengue infection. As systemic immunity and non-canonical inflammasome is thought to be activated by LPS, an increase of LPS in plasma in DWS+ (dengue with symptoms) and SD (severe disease) patients further supports the LPS induced inflammatory responses. This indicates that apart from viral factors, elevation of LPS in plasma also contributed its part to boost cytokine storm along with other danger-associated molecule patterns (DAMPs). Therefore, prediction of biomarkers early in dengue patients fosters proficiently patient triage and permit improved healthcare support for the population specially during dengue outbreaks.

### DENV Induced ADE and Inflammasome

ADE (antibody-dependent enhancement) has been proposed to be one of the most important reasons for cytokine storm. ADE develops when preexisting antibodies from a primary (first) DENV infection, present in the body, bind to an infecting heterologous DENV particle during re-infection. The antibodies from the primary infection do not have neutralizing capacity against the virus, rather they form Ab–virus complex that binds to Fcγ receptors (FcγR) on circulating monocytes. This results in the receptor mediated endocytosis of the Ab–virus complex, further augmenting the dengue infection and resulting in dengue severity (Whitehead et al., 2007; Raj Kumar Patro et al., 2019). During secondary dengue infection, antibody-dependent enhancement (ADE) has been an anticipated mechanism in explaining dengue hemorrhagic fever (DHF; Guzman and Vazquez, 2010) and studies have observed higher IL-1β level in DHF patients as compared to DF patients (Cui et al., 2016; Kamaladasa et al., 2016). A study has demonstrated the ADE triggered cytokines including IL-1β were studied in primary human monocytes utilizing the anti-DENV mAbs from patient. The data demonstrated that DENV-2 infection in monocytes mediated by ADE induces mature IL-1β secretion in 4 h, independent of DENV replication although it requires the stimulation of spleen tyrosine kinase (Syk). Pharmacological blockade of caspase-1 as well as genetic blockade of NLRP3 drastically ablated DENV derived IL-1β secretion (Callaway et al., 2015). Of note, weak NLRP3 inflammasome activation was observed in monocytes in this study as compared to inflammatory macrophages (Wu et al., 2013b), and it may be due to differential expression of CLEC5A in monocytes vs. inflammatory macrophages as later shows significantly very high expression of CLEC5A (Batliner et al., 2011). DENV immune complexes induced activation of Syk can have wider effects as activation of Syk also upregulates the TNF and IL-6 expression. It is important to note that activating ERK1/2 by Syk elevates IL-1β secretion, and inhibition of Syk and ERK1/2 diminished the ADE-triggered IL-1β release (Callaway et al., 2015). In addition to the role of Syk activation during ADE in DENV infection, downstream to C-type lectin receptor activation of SyK activation play a crucial role in activating inflammasome during fungal pathogens and helminths infection (Gross et al., 2009; Poeck and Ruland, 2010; Ritter et al., 2010). In addition, malarial hemozoin upon being engulfed by phagocytes, induces Syk mediated inflammasome activation (Tiemi Shio et al., 2009). This suggest that Syk could be a potential therapeutic focus to restrict the “pathogenic cytokine storm” of severe dengue. Furthermore, a recent study has setup a secondary infection experiment to mimic ADE to demonstrate the immune response of human monocytes to the DENV infection. Human individual monocytes were selected based on their past exposure to severe disease or non-severe dengue and further exposed to each four DENV serotypes subsequent incubation with autologous serum. Results exhibited the marked increased viral load, viral sensing gene expression (NLRP3, RIG-1, IFN-β) and production of inflammatory cytokines, specifically IL-1β in the monocytes of individuals with past severe dengue when compared to monocytes of individuals with past non-severe dengue advising the influence of initial innate immune responses and inflammasome components NLRP3, IL-1β in disease outcome (Kamaladasa et al., 2019).

### Inflammasome and Vascular Leakage

**Figure 1:**
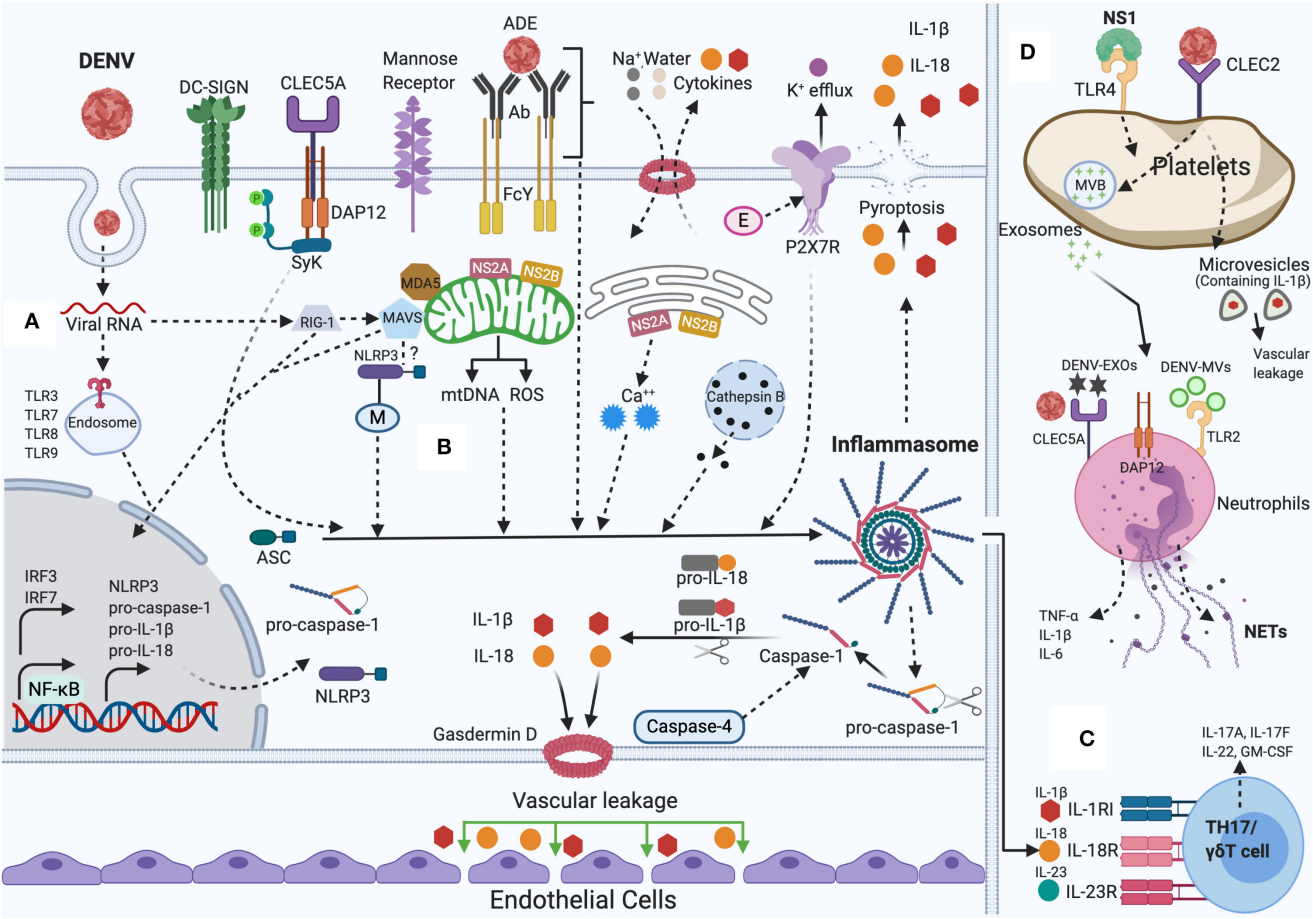
NLRP3 inflammasome activation by DENV: During DENV infection in host cells (Monocytes, Macrophages, Dendritic cells, Endothelial cells, among others). **(A)** Priming (Signal 1): Viral RNA is sensed by cytosolic PRRs (e.g., TLR3, TLR7, TLR8, TLR9, RIG-I, and MDA-5) and triggers the NF-κB signaling, that increases the transcription of pro-caspase-1, ASC, inflammasome receptor (NLRP3), pro-IL-1β, and pro-IL-18. **(B)** Activation (Signal 2): The inflammasome complex is formed and initiated by a secondary stimulus, sensed within the cytosol such as ADE activation, CLEC5A-DAP-12-Syk mediated response, K^+^ efflux, ROS generation, mtDNA release, Cathepsin B release, Ca^++^ mobilization, DENV viral proteins (E, M, NS2A, NS2B) mediated DAMPs. Activated NLRP3 inflammasome stimutates the auto-cleavage of pro-caspase-1. Activated caspase-1 further triggers the proteolytic process of pro-IL-1β, pro-IL-18 into its active form as well as gasdermin D (GSDMD) that induces vascular leakage and cell death (pyroptosis). **(C)** DENV induced IL-1β also stimulates the generation of IL-23 and IL-6. IL-1β, IL-18, along with IL-23 stimulates Th17/γδ T cells to release pro-inflammatory cytokines that mounts adaptive immune responses to DENV infection. **(D)** DENV activates platelets and induces the secretion of EVs (DV-EXOs and DV-MVs), that results in the CLEC5A and TLR2 mediated release of proinflammatory cytokines and NETs formation in neutrophils contributing to vascular leakage during dengue infection. NS1 protein, through autocrine loop triggers TLR4 signaling on platelets and thereby amplifies the platelets mediated inflammasome response to DENV infection. Figure generated with Biorender.com.

Although we have discussed above the role of inflammasome and dengue severity, a recent study demonstrated the mechanism regarding the relationship between augmented inflammatory response and damage of vascular barrier integrity. The study supports that inflammasome induced IL-1β plays a central role in tissue injury and vascular leakage. DENV infection mediated IL-1β activation are found in infected patient blood samples, human peripheral blood mononuclear cells (PBMCs) and monocyte-differentiated macrophages (THP-1) as well in C57BL/6 mice and mouse bone marrow derived macrophages (BMDMs). Evidence demonstrates that IL-1β augments tissue injury and leakage in IFNAR-/- C57BL/6 mice, although IL-1 receptor antagonist (IL-1RA) protects from this injury (Pan et al., 2019b). Together, the collected results reveal the mechanism of DENV pathogenesis and establish the contribution of IL-1β in DENV-associated pathology and recommend the consideration of IL-1RA for effective therapeutics in DENV patients' treatment. The summary of the discussed mechanisms is shown in Figure 1.

### DENV Induced Pyroptosis

Pyroptosis is a programmed cell death (inflammatory) that engages the inflammasomes activation and subsequent caspase-1 activation. Caspase-1 facilitates the processing of pro-IL-1β, pro-IL-18 into the mature IL-1β and IL-18 forms that are released to outside of the cell (Man and Kanneganti, 2016). Caspase-1 further cleaves between C and N domains of Gasdermin D, and the N-terminal domain further attaches to membrane lipids and forms pores that results in cell lysis that facilitates the influx of water molecules leading to cell swelling and subsequent rupture (pyroptosis). Pyroptosis cell death involves the damage of cell membrane integrity and upregulation of the caspase-1 and an upsurge in IL-1β and IL-18 production (Zhao et al., 2018). During dengue infection, apoptosis mediated cell death has been reported, however, evidence has also demonstrated the pyroptosis (inflammatory cell death) in monocytes. Monocytes displayed the activation of caspase-1 and subsequent production of IL-1β accompanied with release of cellular content by 96 h during DENV-2 infection (Tan and Chu, 2013). As we have discussed above, DENV infection induces the inflammasome activation and subsequent cell death. Cell death has been linked to the upregulation of CLEC5A in inflammatory macrophages (Wu et al., 2013a). Of note, upregulation of CLEC5A has been reported in murine monocytes after DENV infection (Cheng et al., 2016), supporting the notion that CLEC5A plays a crucial role in monocyte cell death by pyroptosis. Another study demonstrated a unique mechanism where DENV-2 infection to primary macrophages activates caspase-1 and caspase-4, and subsequent release of IL-1β. Further, loss of cell viability along with the release to the lactate dehydrogenase was observed in extracellular medium. This study demonstrated that caspase-4 activates the caspase-1 and that caspase-4 serves as upstream activator of caspase-1 as inhibitor of caspase-4 ablated both caspases activity and subsequent secretion of IL-1β (Figure 1). This study shows the new role of caspase-4 in activation of inflammasome and pyroptosis during DENV-2 infection (Cheung et al., 2018). This study shows that DENV-2 infection in macrophage results in decreased viability and increased LDH, revealing cell damage and lysis; however, macrophages as beneficial or pathogenic role in cell death remains controversial.

### Dengue Viral Protein Triggers Inflammasome

Several viral proteins have displayed the strong influence on inflammasome activation and elicited the host immune response; as demonstrated in the influenza A virus M2 protein and encephalomyocarditis virus (EMCV) 2B protein (Ichinohe et al., 2010; Ito et al., 2012). The wide range of viral proteins with their modulating effects on inflammasome have previously been discussed in detail (Lupfer et al., 2015; Shrivastava et al., 2016). In the context of DENV, EDIII is regarded as a highly immunogenic protein. In THP-1, EDIII activates NLRP3 inflammasome via NF-κB pathway resulting in caspase-1 activation and subsequent IL-1β and TNF-α secretion. Increased ROS production and potassium was suggested to be the mechanism behind EDIII protein mediated IL-1β production and release (Khan et al., 2019). Moreover, DENV M protein that plays an important role in DENV packaging and egress (Junjhon et al., 2008; Lin et al., 2011), also displays a role in imparting host innate immune response. This study shows that DENV M induces NLRP3 inflammasome and IL-1β release that further induces the endothelial permeability and vascular leakage in mice. More importantly, M protein stimulates tissue injuries in wild-type (WT) mouse tissues, however the effect of M protein was inhibited in NLRP3 knockout (NLRP3^−^/^−^) mouse tissues, indicating a vital role of M protein-stimulated vascular leakage. The effects of M protein in triggering inflammasome activation and IL-1β secretion and subsequent pathological effect is suggested due to the interaction of M protein with NLRP3 (Pan et al., 2019a). However, the molecular mechanism through which M protein binds to NLRP3 is still elusive and warrants more investigation. NS1 is a non-structural protein of DENV and plays a role in DENV replication complex along with other non-structural proteins (Muller and Young, 2013). NS1 is specific in a particular way; this is the only non-structural protein secreted in extracellular milieu (Thiemmeca et al., 2016) and increased NS1 in patient serum is considered as a marker of severe dengue (Libraty et al., 2002). Furthermore, NS1 serves as viral PAMP that triggers TLR4 on the surface of endothelial cells and leukocytes which leads to endothelial dysfunction as well as inflammation (Beatty et al., 2015; Chen et al., 2016; Puerta-Guardo et al., 2016). Recently, NS1 translation and secretion has shown to complement DENV induced inflammasome. The study shows that DENV triggers abortive viral infection in platelets, where DENV translate and replicate its genome without releasing the viral particle. During this infection process, NS1 augments the platelets response via autocrine loop to the DENV infection by caspase-1 mediated secretion of IL-1β and translocating and releasing the stored factors. Notably, NS1 doesn't trigger the direct release of IL-1β, however NS1 triggers the TLR4 on platelets and amplifies the platelets response to DENV, indicating the complex interplay between host and viral factors that leads to the dengue severity (Quirino-Teixeira et al., 2020). In addition, DENV non-structural proteins, NS2A and NS2B have been reported to trigger NLRP3 inflammasome activation. DENV NS2B plays a role in replication, viral protease co-factor and in degradation of cGAS (Aguirre et al., 2017). In addition, NS2B as a protease complex (NS2B/NS3) inhibits mitochondrial fusion by cleaving the mitochondrial fusion proteins i.e., Mfn1 and Mfn2 (Yu et al., 2015). DENV NS2A, is a hydrophobic transmembrane protein with a diverse role in the virus life cycle. NS2A displays a regulatory role in replication, viral assembly, and viral release and most notably by hindering the JAK-STAT and interferon pathways (Leung et al., 2008; Xie et al., 2013, 2015). Moreover, additional findings have demonstrated that DENV-2 NS2B and DENV-2 NS2A as “viroporins” permeabilize host cell membranes (León-Juárez et al., 2016; Shrivastava et al., 2017). Viroporins from several virus that can increase host cell membrane permeability, have shown to activate the NLRP3 inflammasome through various mechanism and thereby regulate antiviral innate immune responses (Guo et al., 2015; Farag et al., 2020). This study initially demonstrated that DENV-2 induces NLRP3 activation, caspase-1 activation and subsequent IL-1β secretion in endothelial cells (HMEC-1). NS2A and NS2B have shown to be co-localized in the endoplasmic reticulum and mitochondria. Furthermore, NS2A and NS2B were sufficient to trigger the activation of NLRP3 inflammasome, and IL-1β secretion through caspase-1 activation in endothelial cells. Pharmacological inhibition of NLRP3, caspase-1 and CRISPR knockout of ASC in endothelial cells showed that NS2A and NS2B mediated inflammasome and IL-1β secretion was NLRP3 and caspase-1 specific. Lastly, the study also suggested that NS2A triggering NLRP3 inflammasome activation is due to Ca^++^ homeostasis and/or mitochondrial disruption /ROS production (Shrivastava et al., 2020). In summary, these evidences show the dengue virus proteins induced mechanism in activating NLRP3 inflammasome. Furthermore, this mechanistic insight with more detailed study would shed more light on DENV pathogenesis understanding (Figure 1). These studies may help in designing and developing new therapeutic candidates against dengue virus.

## Concluding Remarks and Future Perspective

DENV infection comprises immune dysfunction that leads to leakage of vascular fluids and cytokine storm which are suggested as main contributors to the pathogenesis of DENV, culminating in life-threatening hypovolemic shock (Culshaw et al., 2017). Numerous studies have aimed to understand the role of inflammasome in pathogenesis of virus. Inflammasome activation has been accepted as a crucial player in the outcome of DENV infection. Inflammasome induced by DENV facilitates caspase-1 activation and synthesis and secretion of IL-1β and IL-18. Thus, the discussed investigation founded a promising direct linkage between inflammasome activation and dengue severity, while considering the contribution of caspase-1, IL-18 and IL-1β, to DENV-mediated patient pathology. Particularly, IL-1β plays an indispensable role in the severe dengue complex immunopathological condition. Therefore, a question arises, whether therapeutics targeting IL-1β may be useful in the treatment of DENV associated diseases? Inflammasome activation plays a crucial role in controlling numerous pathogens, and thus loss of IL-1β can lead to impaired immune defense. In clinic settings, drugs directed to inhibit NLRP3 or IL-1β are now being utilized for numerous autoimmune diseases, however not enough clinical research has supported the use of such treatments in viral infection. Though, the precise mechanisms underlying DENV induced damage sensing are still debatable, the existing evidence suggests that NLRP3 ligand directly senses the DENV. The precise mechanism of sensing is still elusive and warrants more research. Whether NLRP3 senses the DENV directly or by some other means and if so, how? NLRP3 often acts in concurrence with other inflammasomes sensors such as AIM2 and IFI16, however, whether these ligands sense DENV along with NLRP3 needs further examination. In the context of IL-18 in dengue infection, whether IL-18 confers protective or detrimental role is still unknown. Although pyroptosis might be relevant for viral clearance of infected cells, research regarding the role of DENV-induced pyroptosis is still in a primitive phase. More studies are warranted to understand DENV-induced proptosis in different cell culture models as well as in clinical settings. Mitochondria have been regarded as an important component in inflammasome activation either by ROS generation, mtDNA release, autophagy, or cell death (West et al., 2011; Zhou et al., 2011). Moreover, it has also been demonstrated that mitochondria adapter protein (MAVS) localizes the NLRP3 inflammasome on mitochondria membranes (Subramanian et al., 2013). In DENV infection, mitochondria have also been demonstrated to play a crucial role in regulating host immunity either by ROS generation or by mtDNA release (discussed above in this review). Does DENV induced inflammasome occur on mitochondria membrane and if so, how? Another question is whether DENV viral factors interact directly with mitochondria components? Therefore, more studies are required to unravel the link between DENV infection and mitochondria as it can provide several insights to regulate the inflammatory response during infection. Research should also focus on several host molecules that have been suggested to play a crucial role in regulating the dengue severity such as CLEC5A, TLR2, TLR9, inhibitors of the tyrosine kinases Syk and Btk (discussed above in text). In addition, a deeper understanding of immune evasion molecules evolved by viruses that can inhibit the function of inflammasome will expect to uncover novel concepts and may eventually identify targets in the treatment and prevention of DENV severity. Notably, a better understanding of the equilibrium between detrimental vs. favorable inflammasome activation is also indispensable, as host activation of inflammasome is a “double-edged sword” to defense viral infection. As the systematic vision of the inflammasome rises, prospects to create new therapeutic interventions for patients with DENV severity enhance proportionately. It is important to mention that although inflammasome activation does appear to contribute to dengue, other mechanisms such as an early antiviral response (IFN response), also plays a major contributor along with high levels of immunosuppressive cytokines during dengue virus pathogenesis. Moreover, a different and very strong approach against arbovirus has also been considered by targeting the mosquito vector. DENV virus enters the host along with mosquito saliva at the skin epidermis and dermis level during mosquito bite. To date, various findings have reported the capacity of *Ae. Aegypti* saliva that enable blood feeding, pathogen transmission and regulate host innate and adaptive immune responses through its several pharmacologically active proteins that saliva contains (Pingen et al., 2016, 2017; Wichit et al., 2016; Manning et al., 2018). For example, 34 kDa protein from *Ae. aegypti* was found to prevent the expression of IRF-3, resulting in the inhibition of type I IFN production (Surasombatpattana et al., 2014). Aegyptin protein from *Ae. aegypti*, decreases DENV titers in mice along with increased GM-CSF, IFN-γ, IL-5, and IL-6 concentrations in serum as compared to solitary DENV inoculated to mice, displaying the impacts of Aegyptin in DENV infectivity through activation of the immune response (McCracken et al., 2014). More research with enhanced knowledge regarding the interactions between saliva proteins and primary host immune cells will help to identify the key cell populations/molecules/pathways controlling the infection efficiently. Increased characterization and improved functional understanding of the potential role of saliva proteins on DENV infection could offer unique targets that may support in the development of mosquito vector protein based novel therapeutics and vaccines.

## Author Contributions

GS wrote the manuscript. GS, EC, and PV wrote the final version of the manuscript and approved the submitted version. All authors contributed to the article and approved the submitted version.

## Conflict of Interest

The authors declare that the research was conducted in the absence of any commercial or financial relationships that could be construed as a potential conflict of interest.
